# Loneliness is not associated with attention interference of negative social information: Evidence from four studies

**DOI:** 10.1371/journal.pone.0333167

**Published:** 2025-09-23

**Authors:** Anita Restrepo, Emily M. Silver, Karen E. Smith, Sabina Raja, Frederica Rockwood, Colleen Wohlrab, Kelly E. Faig, Greg J. Norman

**Affiliations:** 1 Department of Psychology, University of Chicago, Chicago, Illinois, United States of America; 2 Department of Psychology, Rutgers University-Newark, Newark, New Jersey, United States of America; 3 Department of Psychology, Hamilton College, Clinton, New York, United States of America; RIKEN CBS: RIKEN Noshinkei Kagaku Kenkyu Center, JAPAN

## Abstract

Extended experiences of loneliness, defined as perceived social isolation, are associated with lasting impacts on health outcomes. One proposed mechanism through which loneliness contributes to health risk is heightened vigilance to cues of social threat resulting in extended activation of stress responses systems. This heightened vigilance is thought to be driven by loneliness-related shifts in a variety of cognitive and affective processes, though the differential effects of loneliness on specific stages of processing remain unclear. The present study examined four datasets using individual participant data meta-analytical techniques to test the link between loneliness and attention interference to social threat cues in an Emotional Stroop task. Despite existing theoretical frameworks predicting heightened attentional interference for negative social information in lonely individuals, we found no support for this effect across the four samples. These findings highlight the need for further work delving into the complex interplay between distinct perceptual processes associated with loneliness and how they contribute to the maintenance of loneliness states over time.

## Introduction

Loneliness, or perceived social isolation, is defined as a perceived discrepancy between an individual’s desired and actual social relationships, in which the individual’s social needs are not being met [1,2]. For a socially interdependent species like humans, having sufficient and high-quality social relationships represents a critical cue of safety [3,4]. Evolutionarily, these relationships are essential and provide access to shared resources, emotional support, and a protective social network [5–7] in the form of friends and family. Thus, loneliness indicates a potential threat to one’s ability to access the benefits of social integration and is associated with heightened and sustained vigilance to cues of threat, particularly social threat [3,5,6]. This vigilance might manifest in a variety of ways, like incorrectly interpreting a friend’s neutral comment as a personal attack or withdrawing from a group conversation due to fear of exclusion. When experienced over prolonged periods of time, loneliness can result in a variety of adverse health outcomes like anxiety, depression, chronic pain, and risk of cardiovascular disease [6,8,9]. A primary mechanism linking loneliness and health is chronic threat of and hypervigilance to negative social evaluations and associated threats to social integration [10–13]. A state of hypervigilance is characterized by the activation of multiple stress response systems preparing individuals to cope with threats. Continuous activation of these systems linked to chronic loneliness is one potential mechanism that can increase pathology risk [14,15]. Though great strides have been made, work is needed to further understand the specific cognitive and affective mechanisms, such as initial attentional and perceptual processes, underlying loneliness-related shifts towards hypervigilance to threat. Identifying the specific mechanisms through which loneliness contributes to hypervigilance to threat can enable the development of more effective and targeted interventions for individuals experiencing chronic loneliness and associated health outcomes. Here, we combine four datasets to investigate how attentional interference specific to negative social information, one aspect of early perceptual processing with implications for hypervigilance, is affected by loneliness.

### Social information processing and loneliness

Loneliness-related hypervigilance directed towards the detection of potential social threat is facilitated by shifts towards enhanced processing of social and emotional information. Lonely individuals differentiate social threat images faster than non-lonely individuals [16], show stronger activation of the visual cortex when viewing unpleasant social images [17], and demonstrate biases in sensitivity to, and accuracy of, detection of emotions – particularly negative emotions [18–21]. Loneliness is also associated with higher sensitivity to social rejection and increased hostility to ambiguous social threat in children [22] and in adults [23,24], suggesting that cognitive-affective shifts in lonely individuals promote attunement to potential social threats. Additionally, lonely individuals demonstrate increased recall of social information, which is associated with increased attention to emotional vocal tones and discrimination of emotional facial expressions [25]. Together, this evidence suggests that loneliness shifts affective and cognitive processing towards the prioritization of social information in general and social threat in particular, a strategy that can facilitate the avoidance of negative interactions, but can also result in inappropriate reactions, like preemptively responding to a friendly joke with a defensive rebuttal.

### Loneliness and attention

Attention, or the targeted allocation of cognitive resources to subsets of incoming sensory stimuli in order to facilitate and direct perception [26], is one of the many cognitive and affective mechanisms involved in loneliness [13]. Changes in attention can have important effects on subsequent emotional and cognitive processes [27–30], including those, like memory [25] and appraisal of social information [24,31], that show shifts in response to loneliness. Empirical studies provide evidence for attentional shifts in loneliness. For example, eye-tracking studies suggest that lonely adults are faster to fixate on socially threatening stimuli [18,32] and demonstrate longer viewing times of scenes showing individuals who are being socially rejected [18]. In addition, loneliness is associated with more difficulty disengaging attention from socially rejecting stimuli in children [22]. This evidence suggests loneliness shifts attentional processes to make individuals more vigilant to information that communicates potential threat, which may help to maintain feelings of loneliness over time [10], and subsequently contribute to the development of negative psychosocial and health effects.

Attentional interference occurs when salient task-irrelevant stimuli compete with task-relevant stimuli for cognitive resources, thus interfering with an individual’s ability to adequately perform task-related goals. This interference results in a need to exert additional cognitive effort to remain focused on the task-relevant stimuli [33]. If threat-related information is highly salient to an individual, they should demonstrate more attentional interference effects when presented with the threatening stimuli than with non-threatening stimuli. For example, lonely individuals may be more distracted by the singular frowning face in the audience when giving an oral presentation instead of focusing on the many smiling faces. As such, heightened attentional interference in the presence of potential threat in lonely, as compared to less lonely, individuals are likely indicative of a state of sustained vigilance. Indeed, past work using an auditory emotional Stroop task shows lonely individuals demonstrate greater attentional interference, as indexed by slower reaction times, in response to words presented verbally when these are spoken in a negative vocal tone, a cue of potential social threat [34]. It is unclear, though, if and in what contexts, loneliness-related hypervigilance prioritizes exclusively social and/or exclusively negative information. For example, lonely individuals do not demonstrate a bias towards social stimuli (faces) vs. non-social stimuli (houses) in an automatic attention paradigm [35] and loneliness does not shift visual attention to social cues [36]. In sum, while the empirical work suggests attentional interference does occur in lonelier individuals, a more robust understanding of the generalizability of these effects as well as the conditions under which the specific targets of sustained vigilance vary is needed. One way to address this gap is to incorporate data from multiple samples and study designs to assess whether any effects of loneliness on attention remain consistent across multiple contexts. The Color Stroop Task is a foundational, well-established attention interference task, and its emotional variant – the Emotional Stroop task – has been widely and effectively used in many participant populations [37–40] to examine attention interference resulting from specific emotionally charged semantic meaning [37,41]. If lonely individuals demonstrate hypervigilance to cues of social threat, then the negative social semantic meaning of specific words should be particularly salient for these lonely individuals. As such, assessing effects of loneliness on attention interference utilizing a long-standing experimental paradigm represents a strong step towards disentangling the complex relationships between loneliness and subprocesses of attention.

### The current study

In the current study, we tested whether loneliness influenced attention interference effects for negative social information. We combined data from four separate studies that utilized the Emotional Stroop paradigm, a widely used and validated tool that assesses domain-specific attention interference effects [37]. The four samples offer a diversity of participant ages and experimental formats, including in-person administration of the Emotional Stroop as well as an online paradigm. Based on the theorized role of attention changes in generating hypervigilance to social threat in loneliness, we expected individuals with high loneliness to exhibit increased attention interference effects—reflected by slower reaction times—for negative social words on the Emotional Stroop as compared to other word categories.

## Materials and methods

The current work combined four separate datasets (total N = 414) to test the relationship between loneliness and attention to cues of social threat in an Emotional Stroop paradigm. The datasets were collected as part of larger studies that sought to address unrelated questions (see S1 File), but all included identical measures of loneliness and modified versions of the Emotional Stroop task. The four studies were selected for inclusion based on the shared availability of these measures. All studies were approved by the University of Chicago Institutional Review Board (IRB #s: 13–1435, 16–0812, 17–1510, 21–1111). Participant recruitment across the four studies started on February 5^th^ of 2014 and ended on March 14^th^, 2022. For all studies, participants provided written informed consent prior to participation and were debriefed following the experiment. Table 1 shows subject-level descriptive statistics for each study and S1 Table in S1 File shows the differences between the study paradigms.

**Table 1:** Sample demographics.

Dataset	N	Gender		Age		Race					Loneliness	
		Female	Male	Mean	SD	White	Black	Hispanic	Asian	Other	Mean	SD
Study 1	142	76	63	42.35	12.61	118	7	6	7	1	38.75	13.85
Study 2	76	35	41	39.92	10.36	52	10	5	7	2	42.66	14.41
Study 3	83	28	50	37.99	9.52	59	7	5	7	1	43.89	14.84
Study 4	113	77	36	20.02	1.99	39	6	16	41	11	40.83	9.25

Descriptive statistics of the samples from each of the four studies.

### Questionnaire measures

#### Loneliness.

Across all studies, loneliness was measured using the 20-item UCLA Loneliness Scale – Version 3 [42], a widely used loneliness questionnaire [43] that was originally validated on a diverse sample of college students, nurses, teachers, and elderly individuals [42]. Questions are rated on a four-point scale (1 = Never, 2 = Rarely, 3 = Sometimes, 4 = Often). Sample items include “How often do you feel alone?” and “How often do you feel isolated from others?”. Scores are summed, and higher total scores indicate higher levels of loneliness. Cronbach’s alphas for all studies were ≥ 0.92 (Study 1: 0.96, Study 2: 0.96, Study 3: 0.97, Study 4: 0.92) and McDonald’s omegas for all studies were ≥ 0.74 (Study 1: 0.78, Study 2: 0.74, Study 3: 0.75, Study 4: 0.75).

#### Depression.

Given established correlations between loneliness and depression [44] and the presence of the same depression scale in all four studies, exploratory analyses involving depression were also included. Symptoms of depression were measured using the Center for Epidemiological Studies Depression (CES-D) Scale [45] in all four datasets. The CES-D is a short survey designed for assessment of depressive symptomatology in the general population that was originally validated in both clinical and non-clinical populations [45]. Sample items include “I felt that everything I did was an effort” and “I felt depressed”. Consistent with prior work [46–48], item 14 (“I felt lonely”) was removed from models using the scale to avoid overlap with the loneliness measure (see S1 File for results utilizing the CES-D). Cronbach’s alphas for all studies were ≥ 0.89 (Study 1: 0.93, Study 2: 0.94, Study 3: 0.96, Study 4: 0.89) and McDonald’s omegas for all studies were ≥ 0.74 (Study 1: 0.78, Study 2: 0.80, Study 3: 0.86, Study 4: 0.74).

### Emotional stroop task

**Fig 1:**
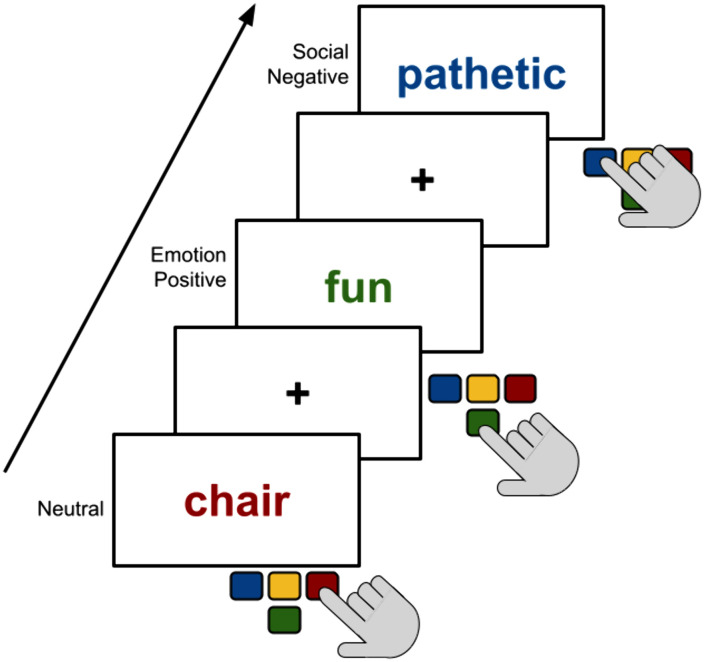
Emotional Stroop paradigm. Participants saw a fixation cross followed by a single word written in red, blue, green, or yellow ink. The semantic meaning of the words was from one of four categories: Neutral, Emotion Positive, Emotion Negative, Social Positive, or Social Negative. Participants were tasked with pressing on the keyboard button that represented the ink color of the word as fast and as accurately as possible.

All participants performed a variation on the Emotional Stroop task [37]. Participants were asked to identify the ink color of the word shown on the screen as quickly as possible by pressing specified keys on the keyboard (Fig 1). Ink colors consisted of blue, red, green, and yellow. All studies included at least five stimuli categories: neutral, emotion-positive, emotion-negative, social-positive, and social-negative (see S1 File for the exact word lists by study). Categories were determined based on the semantic content of the words (positive vs. negative valence and social vs. non-social content). There were minor differences between the tasks for each study which can be found in Table A in S1 File. Across all studies, participant accuracy was high, with 98% of trials answered correctly (i.e., the ink color was correctly identified).

### Statistical analysis

The four studies were combined using a one-stage individual participant data (IPD) meta-analysis framework following recommendations from Riley, Tierney, and Stewart [49] in order to aggregate across all data available while still accounting for variability resulting from differences in data collection in the four studies. We chose to use a one-stage as opposed to a two-stage IPD meta-analytic strategy due to recommendations for studies with small samples. A one-stage approach minimizes the chance that underlying modeling assumptions are incorrect since this strategy models the actual data instead of modeling aggregated effect sizes [49]. We ran three-level hierarchical linear models for reaction times, with trials nested within subjects nested within study. Given the low variability in reaction times at the level of study (2%) compared to across subjects within studies (26%), we included random intercepts and no random slopes. For the main model, we included fixed effects for word category (i.e., neutral, emotion-positive, emotion-negative, social-positive, social-negative), loneliness score (grand-mean centered), and the two-way interaction between these variables. For the Color Stroop test, we included a fixed effect for trial congruency (binary) with no fixed effects for loneliness or word category. All models included only accurate trials where reaction times were not zero. Additional models with differing reaction time outlier removal techniques are reported in S1 File. Simple-slopes analyses were run using the *emtrends* function from the *emmeans* package [50] in order to assess interactions involving categorical variables (e.g., stroop category).

## Results

In contrast to our hypothesis that loneliness would be associated with increased attentional interference for negative social information, there were no significant effects for either loneliness (*Χ*^*2*^(1) = 0.44, *p* = 0.51) or the interaction between loneliness and word categories (*Χ*^*2*^(4) = 3.82, *p* = 0.43) on reaction times. Due to the non-significant test for word category overall, pairwise contrasts between specific word categories are not reported. We also ran models using different outlier techniques following common strategies found in previous work [36,46,47], which can be found in S1 File. These models were consistent with there being minimal evidence for an effect of loneliness on attentional interference. We implemented exploratory sensitivity analyses using models that included additional covariates at both the subject and study level to explore whether these variables influenced any effects of loneliness on the Emotional Stroop. These variables included participant gender, age, ethnicity, and symptoms of depression, as well as study characteristics. Across these sensitivity analyses, which are reported in S1 File, we found minimal evidence for a consistent relationship between loneliness and Emotional Stroop performance. We also found little support for interactions between loneliness and any additional covariates in predicting Emotional Stroop effects.

To confirm the Emotional Stroop task accurately captured attention interference effects across the different studies, we tested the traditional Stroop effect, i.e., whether reaction times were faster for congruent trials (where the ink color matched the word) as opposed to incongruent trials (where the ink color did not match the word). Of the four studies, three included a traditional color Stroop sequence. Confirming the traditional Stroop effect, trial congruency significantly predicted reaction times (*Χ*^*2*^(1) = 251.03, *p* < 0.001) where reaction times were significantly faster in congruent compared to non-congruent trials (*β *= −106.50, SE = 6.72). This indicates the Stroop task was adequately implemented in these samples and suggests any null findings are not due to incorrect implementation of the task.

## Discussion

Loneliness has consistently been associated with hypervigilance to cues of threat, especially threats to belonging [5,51]. Shifts in attention, like those in sustained social vigilance, impact early stages of information processing [30] and can have significant downstream effects on other cognitive and affective processes. In the current study we examined four separate datasets to investigate whether loneliness is associated with increased attentional interference –a phenomenon resulting from the presence of salient stimuli that compete for cognitive resources–for social threat information using an Emotional Stroop task. Difficulty disengaging from potentially threatening social information should result in attention interference effects. Therefore, increased attention interference in lonelier individuals would be indicative of the presence of hypervigilance to social threat, one of multiple potential mechanisms thought to contribute to chronic experiences of loneliness. However, contrary to predictions, we did not find evidence that loneliness impacted attentional interference for social information.

### Theoretical implications

One explanation of the findings in the current study is that loneliness affects threat perception via changes to other processing stages that do not involve the immediate and subliminal interference effects tested by the Emotional Stroop task. Given that attention is a broad phenomenon encompassing multiple subcomponents, including alerting, orienting, and executive control [52], effects of loneliness likely differ across these subcomponents. In addition, which subcomponents of attention are affected by loneliness might also vary in different populations given how external factors like parenting, early life stress, and social learning environments can shape the development of these constructs during childhood [53,54]. Indeed, some work shows that out of four subcomponents of executive control (social cognition/processing speed, planning/working memory, divided attention/inhibition control, and sustained attention/motor inhibition), only planning/working memory are associated with loneliness in healthy older adults [55]. In contrast, visual attention studies with young adults and children suggest loneliness affects the speed and duration of visual fixation on social threat stimuli [18,22,32], which may involve different underlying attentional processes like alertness, orienting, and sustained focus. While we provide some exploratory sensitivity analyses assessing the role of demographic variables, like gender and age, on loneliness effects in S1 File, we are unable to draw robust conclusions from these analyses given the small number of studies involved (n = 4). Nonetheless, we hope they may provide a launching-off point for future work investigating how loneliness may be modulating specific cognitive subcomponents in different populations, especially in children where these alterations in early stages of development may be particularly impactful.

Some evidence suggests that the sensory modality in which cues of potential threat are presented may also play a role in determining the presence and strength of loneliness effects on cognitive and affective processes. Indeed, a previous study utilizing an auditory version of the Emotional Stroop did find the expected loneliness effects on negative social information as conveyed by increased attention interference to negative vocal tone [34]. In this study, negative social cues were conveyed through the vocal tone of the speaker as they read the Emotional Stroop words to participants. The authors found that loneliness was associated with increased attention interference to words spoken in a negative vocal tone compared to a positive vocal tone, with stronger effects for words that have social semantic meanings [34]. In contrast, an EEG study using the visual Emotion Stroop found no behavioral differences in reaction times based on loneliness [56], but did report significant differences in neural markers for differentiation between social and non-social words; lonely individuals demonstrated brain microstates indicative of differentiation 200ms earlier than less lonely participants [56]. This suggests effects of loneliness may manifest at specific perceptual processing stages depending on characteristics of the information presented (e.g., sensory modality). Additionally, the social relevance of the negative words might be highlighted when heard spoken by an individual (as in the auditory Stroop task) as opposed to simply seen written on a screen.

A related possibility is that the words used in the Emotional Stroop task do not on their own generate sufficiently potent affective responses in participants, and simple reading of the words on the screen is an insufficient emotional stimulus. However, the Emotional Stroop task has been widely and effectively used in many participant populations [37–40], suggesting it reliably elicits adequately valenced emotional reactions. Future research using tasks with more evocative (e.g., pictures, videos), personally meaningful (e.g., autobiographical), and ecologically valid (e.g., interpersonal interactions) stimuli that generate stronger affective responses can provide enhanced insight into how loneliness exerts effects on attention. In addition, it is possible that different subcomponents of loneliness (e.g., social loneliness vs. emotional loneliness) have varying effects on attention, which might be particularly relevant for the Emotional Stroop, where stimuli are selected for their social and emotional content. In line with this, it is possible that results would have differed if a scale other than the UCLA had been used to measure loneliness, particularly if effects on attention interference are specific to certain subtypes of loneliness that are unmeasured or undifferentiated by the UCLA. Nonetheless, given the overlap in the primary latent factor measured across loneliness scales [57], it is less likely that meaningful effects would differ by scale. In sum, more targeted work is needed to disentangle the effects of loneliness on different cognitive and affective processes and how these come together to influence threat perception for lonely individuals.

Effects of loneliness on attention are likely also modulated by the degree of ambiguity in the information presented, defined as information that lacks a single, clear, primary interpretation [58]. For example, recent work suggests that loneliness effects on stimulus perception may depend on levels of ambiguity where lonely individuals interpret ambiguous situations as more negative [23,24]. This is consistent with work demonstrating that hypervigilance associated with anxiety is sensitive to ambiguity [59]. As such, it is possible that hypervigilance to cues of social threat in loneliness is strongest (and most apparent) for ambiguous stimuli. However, the Emotional Stroop words are designed to have a clear primary interpretation and as such are unambiguous. In other words, the words used in the task are clearly valenced and contain either social or non-social information, which did not allow for examination of ambiguity in the current study. As such, the minimal levels of ambiguity in this stimulus set may be partially responsible for the lack of effects of loneliness in the current study. Future work could explore influences of individual experiences on attention to stimuli by obtaining affective ratings of Emotional Stroop words from participants. Beyond this, future studies should investigate the effects of ambiguity of social stimuli on attention interference to determine how this kind of uncertainty contributes to shaping responses in lonely individuals.

Finally, we are confident that the Emotional Stroop task implemented here was accurately indexing attention interference effects given that participants in the samples did demonstrate the traditional color Stroop interference effect. In addition, the high accuracy rate suggests participants were attentive to the task at hand as opposed to clicking keys in a random fashion. Another potential interpretation—as some prior work suggests—is that attentional effects in loneliness may not be specific to negative social information, but instead focus on social information overall [25], or just on negatively valenced information [60,61], regardless of its social content. Nonetheless, across our four datasets there was no indication that loneliness shifted attention towards either social information overall or negative information overall as compared to neutral stimuli. As such, it is unlikely that these alternate proposed effects explain why the current study did not find evidence for an association between loneliness and hypervigilance to specifically social threat. In sum, these findings suggest that the nature of attentional effects in loneliness—particularly those related to interference from social threat cues—might not be as apparent as previously assumed.

To our knowledge, this is the first report to combine multiple Emotional Stroop datasets exploring effects of loneliness on shifts in attentional interference, enabling us to assess the generalizability of any findings. Specifically, the combined sample spanned a wide range of participant ages and demographics, as the datasets represented data collected both online using Amazon’s Mechanical Turk as well as in the lab. The long data collection window (2014–2022) also provides a sampling from different time periods where global events like the COVID pandemic or cultural shifts may have influenced general levels of loneliness. We are confident the lack of an association between loneliness and hypervigilance to social threat in the current study is not due to low-quality behavioral data or a lack of statistical power given the validity of the color Stroop findings, the high accuracy rate in participant responses to the Emotional Stroop, and the large combined sample size. This lack of an association has relevant implications for our theoretical understanding of loneliness and its treatment and prevention. Hypervigilance to negative social information is proposed to be one mechanism that helps to maintain high levels of loneliness, thus leading to chronic loneliness conditions [10]. If, as our findings suggest, the effects of loneliness on attentional processes are more narrow than previously assumed, this implies additional room for interventions that prevent cycles of chronic loneliness and subsequent pathological effects. For example, it may be fruitful for intervention approaches to focus on other aspects of perceptual processing of social information in loneliness, like the reappraisal of social information after it has been interpreted, as opposed to the attentional processes involved in its initial perception. Further work is needed to explore the complexity of the effects of loneliness on attention and cognition via more targeted experimental studies that can disentangle the circumstances—including stimulus characteristics, aspects of the population being studied, and underlying cognitive subcomponents involved—that give rise to specific attentional biases in loneliness.

In addition, while the current study focused narrowly on one subcomponent of attention that is relevant to loneliness, loneliness is a multifaceted construct with many additional characteristics that are likely also involved in shaping cognitive outcomes. For example, social exclusion and loneliness have been associated with changes in neural processing of reward cues [62,63], maladaptive emotion regulation strategies like increased rumination, catastrophizing, and expressive suppression [64,65], as well as altered perceptions of social support given to [66], and received from [67], their close others. As such, hypervigilance to threat is one of a myriad of constructs that influence how lonely individuals perceive incoming social information and their subsequent behavior, underscoring the complex interplay between different cognitive and affective characteristics of loneliness.

## Conclusion

Overall, this study provides insight into the complexity of the relationship between loneliness and attention, which is a potential mechanism through which loneliness biases processing of social information. Our findings do not support the idea that loneliness is associated with attentional interference specific to negative social information, suggesting that other factors involved in information processing—including alternative attention-related processes that are not reflected in the Emotional Stroop paradigm--contribute to loneliness-related biases towards social threat. As such, there is a need for more work investigating the multifaceted effects of loneliness on affective and cognitive processing. Specifically, studies should continue to disentangle the effects of loneliness on distinct substages of information processing by targeting components like memory, appraisal, and other subcomponents of attention and by exploring how different stimulus characteristics may modulate these effects. Ultimately, these empirical findings contribute to the field’s growing understanding of how attentional processes are involved in shaping social perception in loneliness and the specific subcomponents associated with hypervigilance to social threat in lonely individuals. An empirically-supported account of how these cognitive and affective processes change in loneliness is needed to identify narrow targets for intervention to prevent the toll of chronic loneliness on individual health risk and societal wellbeing.

## Supporting information

S1 File Additional study-specific information and analyses. (PDF)
