# Supplementary material for: Loneliness is not associated with attention interference of negative social information: Evidence from four studies
Source: PLoS One. 2025 Sep 23;20(9):e0333167. doi: 10.1371/journal.pone.0333167 (PMC12456806; doi:10.1371/journal.pone.0333167)
Supplement: S1 File — (PDF) [file pone.0333167.s001.pdf]

S1 Supplementary Materials

Data Source:

Data were gathered from four separate studies that sought to answer questions regarding effects of loneliness on cognitive performance, effects of loneliness on feelings of disgust and aversion to illness cues, interactions between loneliness and acute social rejection, and effects of disease cue exposure on cognition.

Alternative Outlier Techniques:

We ran two additional models with different reaction time outlier removal techniques to assess whether the lack of significant findings could be attributed to outlier trials. In the first model, we removed trials with reaction times below 200ms or above 1600ms. In the second model, we retained the 200ms lower cut-off but modified the upper bound to remove trials where reaction times were more than three standard deviations above the participant's mean reaction time. In the first model, we still saw no main effect of loneliness ($X^2(1) = 1.23$, $p = 0.27$) or any interaction with word category ($X^2(4) = 8.67$, $p = 0.07$). In the second model, there was still no effect of loneliness ($X^2(1) = 0.8$, $p = 0.37$) but the interaction with word category became significant ($X^2(4) = 9.88$, $p = 0.04$). Despite this, simple slopes analysis showed that none of the simple slopes for the effects of loneliness significantly differed from zero for any of the word categories. Similarly, pairwise comparisons of the simple slopes by word categories showed that no two categories significantly differed in the slope estimates for loneliness after using a Tukey correction for multiple comparisons. Given the unreliable nature of the significant interaction, we conclude it most likely to be a spurious association.

Models with Covariates:

At the subject level, we found significant moderation of loneliness effects by gender ($p = .000498$), age ($p = .0376$), ethnicity ($p = .0057$), and depression scores ($p = .0095$). Nonetheless, subsequent simple slopes analyses did not find that any of the slopes for the individual associations between loneliness and reaction time at different levels of moderating variables were significant. This indicates no strong conclusions can be drawn about the potential moderating effects of these subject-level variables. At the study level, we found significant moderation of loneliness effects by

several factors: 1) whether or not the study contained a manipulation of some kind (*p* = .0036), 2) the trial length (*p* = .0018), 3) the number of trials included in each block (*p* = .015), and 4) whether the study employed a fixed block order (*p* = .0009). Once again, subsequent simple slopes analyses indicated that individual trend lines for associations between loneliness and reaction times were not significant for the moderation by manipulation or number of trials per block. Nonetheless, some trend lines were significant for the moderation of loneliness effects by trial length and fixed word order. Given the small number of studies included (N = 4), we cannot draw strong conclusions about the directionality or nature of any of these potential moderation effects. Nonetheless, these preliminary analyses indicate further research is needed to determine whether relationships between loneliness and reaction time in the Emotional Stroop are dependent on these specific subject and task-based characteristics.

Table 1

**Dataset**	**Run Online**	**Included Manipulation**	**N Blocks**	**Trial Length (ms)**	**Block Presentation Order**	**N Trials per Block**	**Word Order Randomization**	**Included Color Stroop**	**Had Other Stroop Categories**	**Repeated Words**	**Fixed Block Presentation**
Study 1	✓		6	5,000	random	30	per block with replacement	✓		✓	
Study 2	✓	✓	5	2,000	emo_pos, soc_neg, neutral, soc_pos, emo_neg	40	per block with replacement			✓	✓
Study 3	✓	✓	6	2,000	emo_pos, soc_neg, neutral, soc_pos, emo_neg, color	20	per block without replacement	✓			✓
Study 4		✓	7	3,000	random	20	per block without replacement	✓	✓		

Summary of paradigm differences between the four studies.

Word Lists per Study

Study 1:

Emotion Negative	Emotion Positive	Social Negative	Social Positive	Neutral
1 depressed<br>2 agony<br>3 misery<br>4 anxious	1 lucky<br>2 comfort<br>3 bliss<br>4 hope	1 unwanted<br>2 deceived<br>3 disliked<br>4 humiliated	1 loyal<br>2 faithful<br>3 kiss<br>4 friendly	1 taxi<br>2 chair<br>3 paper<br>4 kettle

5 pain	5 cheer	5 pathetic	5 charming	5 table
6 angry	6 pleasure	6 lonely	6 kind	6 locker
7 frustrated	7 success	7 inferior	7 desired	7 foot
8 sad	8 terrific	8 rejected	8 liked	8 cabinet
9 afraid	9 enjoy	9 betrayed	9 belong	9 square
10 stress	10 pleased	10 insulted	10 devoted	10 bowl
11 irritated	11 happy	11 defeated	11 giving	11 statue
12 panic	12 laugh	12 excluded	12 loved	12 chin
13 anguish	13 delight	13 hostile	13 included	13 board
14 fear	14 joyful	14 inadequate	14 accepted	14 street
15 brutal	15 prize	15 ridiculed	15 admired	15 seat
16 distress	16 brave	16 abused	16 caring	16 barrel

Study 2:

Emotion Negative	Emotion Positive	Social Negative	Social Positive	Neutral
1 depressed	1 lucky	1 unwanted	1 flirt	1 taxi
2 agony	2 comfort	2 deceived	2 belong	2 chair
3 misery	3 bliss	3 disliked	3 included	3 paper
4 anxious	4 hope	4 humiliated	4 charming	4 kettle
5 pain	5 cheer	5 pathetic	5 faithful	5 table
6 angry	6 pleasure	6 lonely	6 devoted	6 locker
7 frustrated	7 success	7 inferior	7 desired	7 foot
8 sad	8 terrific	8 rejected	8 liked	8 cabinet
9 afraid	9 enjoy	9 betrayed	9 affection	9 square
10 stress	10 pleased	10 insulted	10 thoughtful	10 bowl
11 irritated	11 happy	11 defeated	11 giving	11 statue
12 panic	12 laugh	12 excluded	12 loved	12 chin
13 anguish	13 delight	13 hostile	13 kind	13 board
14 fear	14 joyful	14 inadequate	14 accepted	14 street
15 brutal	15 prize	15 ridiculed	15 admired	15 seat

16 distress	16 brave	16 abused	16 caring	16 barrel
17 insecure	17 useful	17 selfish	17 kiss	17 hairpin
18 greed	18 achieve	18 shamed	18 loyal	18 pencil
19 corrupt	19 fun	19 assaulted		19 item
20 cruel				

Study 3:

Emotion Negative	Emotion Positive	Social Negative	Social Positive	Neutral
1 afraid	1 achieve	1 abused	1 accepted	1 barrel
2 agony	2 bliss	2 assaulted	2 admired	2 board
3 angry	3 brave	3 betrayed	3 affection	3 bowl
4 anguish	4 bright	4 deceived	4 belong	4 cabinet
5 anxious	5 cheer	5 defeated	5 caring	5 chair
6 brutal	6 comfort	6 disliked	6 charming	6 chin
7 corrupt	7 delight	7 excluded	7 desired	7 foot
8 cruel	8 enjoy	8 hated	8 devoted	8 hairpin
9 depressed	9 fun	9 hostile	9 faithful	9 item
10 distress	10 happy	10 humiliated	10 flirt	10 kettle
11 fear	11 hope	11 inadequate	11 friendly	11 locker
12 frustrated	12 joyful	12 inferior	12 giving	12 nonchalant
13 greed	13 laugh	13 insulted	13 included	13 paper
14 insecure	14 lucky	14 lonely	14 kind	14 pencil
15 irritated	15 pleased	15 pathetic	15 kiss	15 seat
16 misery	16 pleasure	16 rejected	16 liked	16 square
17 pain	17 prize	17 ridiculed	17 loved	17 statue
18 panic	18 success	18 selfish	18 loyal	18 street
19 sad	19 terrific	19 shamed	19 party	19 table
20 stress	20 useful	20 unwanted	20 thoughtful	20 taxi

Study 4:

Emotion Negative	Emotion Positive	Social Negative	Social Positive	Neutral

1 panic	1 enjoy	1 insulted	1 accepted	1 square
2 anxious	2 joyful	2 hated	2 loyal	2 pencil
3 brutal	3 terrific	3 lonely	3 giving	3 seat
4 greed	4 bliss	4 excluded	4 party	4 bowl
5 stress	5 useful	5 rejected	5 included	5 chair
6 anguish	6 success	6 selfish	6 kiss	6 board
7 cruel	7 comfort	7 ridiculed	7 flirt	7 foot
8 depressed	8 bright	8 shamed	8 belong	8 paper
9 irritated	9 prize	9 pathetic	9 charming	9 statue
10 agony	10 pleasure	10 abused	10 thoughtful	10 street
11 pain	11 happy	11 unwanted	11 devoted	11 cabinet
12 misery	12 delight	12 deceived	12 liked	12 locker
13 distress	13 fun	13 inadequate	13 caring	13 nonchalant
14 sad	14 cheer	14 assaulted	14 loved	14 hairpin
15 insecure	15 lucky	15 humiliated	15 admired	15 table
16 fear	16 brave	16 disliked	16 faithful	16 kettle
17 frustrated	17 pleased	17 defeated	17 friendly	17 taxi
18 corrupt	18 laugh	18 hostile	18 affection	18 item
19 angry	19 hope	19 betrayed	19 kind	19 barrel
20 afraid	20 achieve	20 inferior	20 desired	20 chin
